# Supplementary figures and images for: What are the risk factors of hospital length of stay in the novel coronavirus pneumonia (COVID-19) patients? A survival analysis in southwest China
Source: PLoS One. 2022 Jan 14;17(1):e0261216. doi: 10.1371/journal.pone.0261216 (PMC8759704; doi:10.1371/journal.pone.0261216)

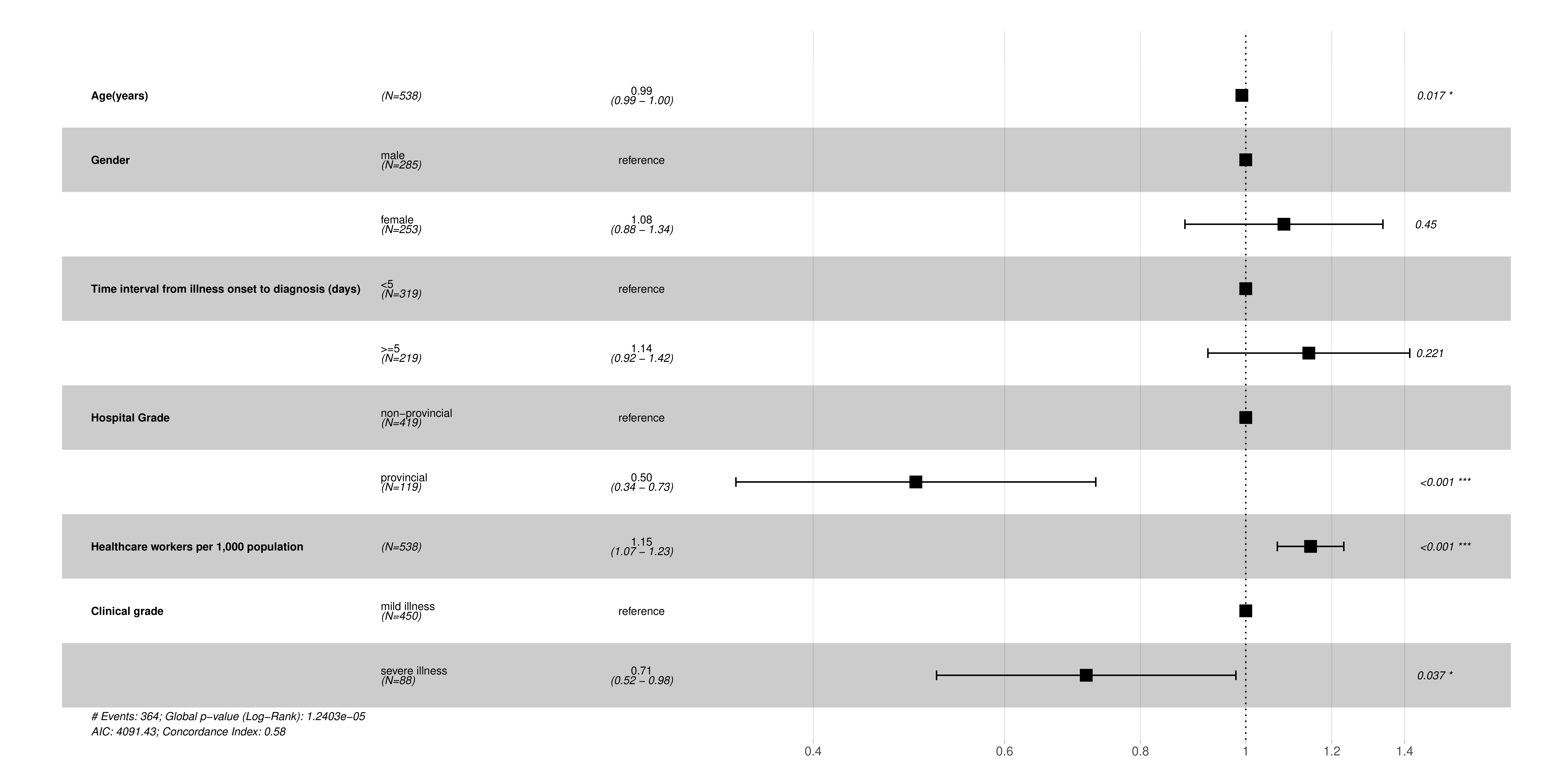

Supplement: S1 Fig — (TIFF) [file pone.0261216.s001.tiff]

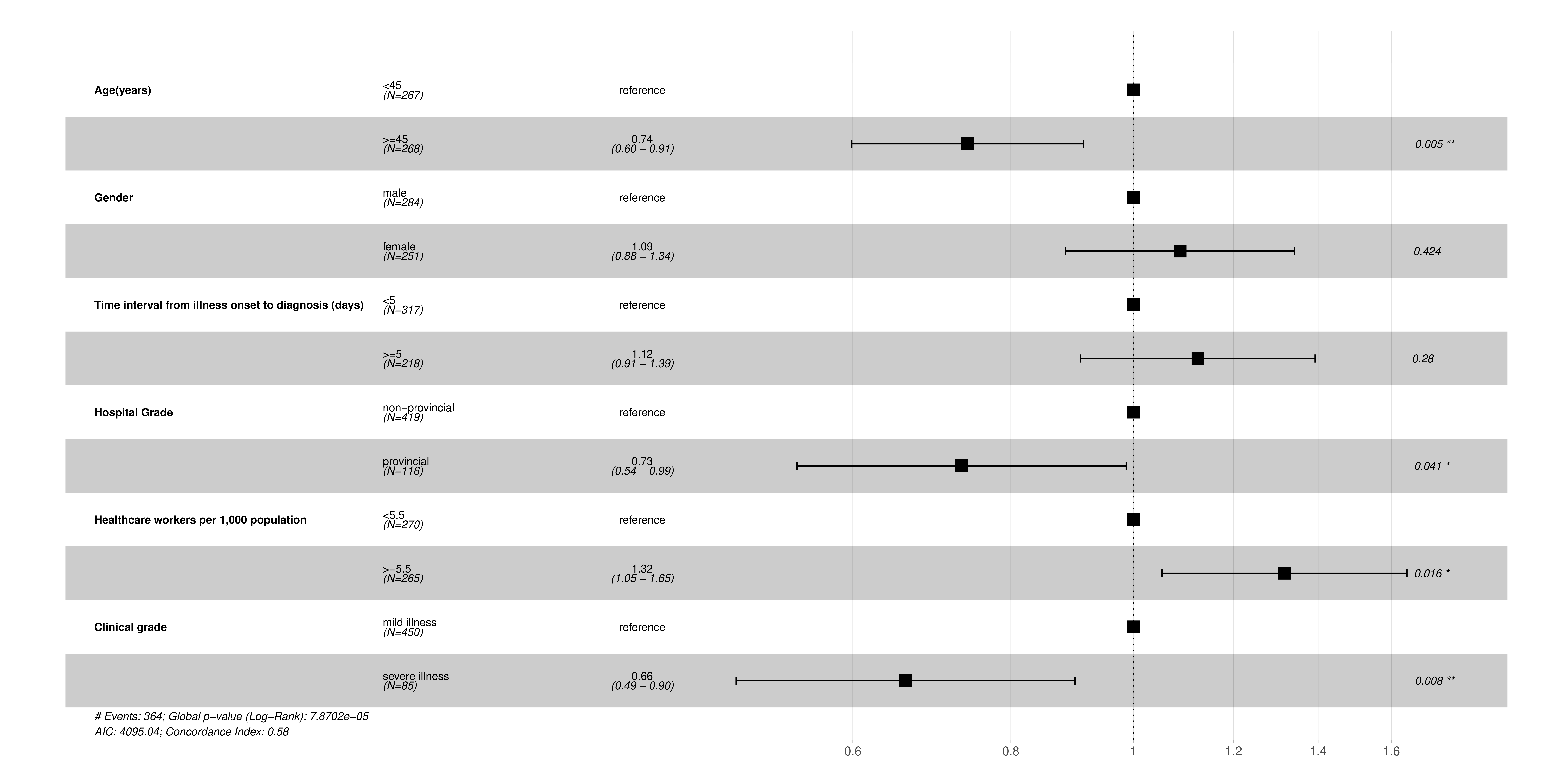

Supplement: S2 Fig — (TIFF) [file pone.0261216.s002.tiff]
